# The inverted cup device for blood transfer on malaria RDTs: ease of use, acceptability and safety in routine use by health workers in Nigeria

**DOI:** 10.1186/s12936-018-2173-0

**Published:** 2018-01-15

**Authors:** Sandra Incardona, Magoma Mwancha-Kwasa, Roxanne R. Rees-Channer, Audrey Albertini, Joshua Havumaki, Peter Chiodini, Wellington Oyibo, Iveth J. Gonzalez

**Affiliations:** 1 Foundation for Innovative New Diagnostics, 9 Chemin des Mines, 1202 Geneva, Switzerland; 2 Hospital for Tropical Diseases, Mortimer Market, London, WC1E 6JB UK; 3 University of Michigan, 500 S. State Street, Ann Arbor, MI 48109 USA; 4 Tropical Disease Research Laboratory, Department of Medical Microbiology and Parasitology, College of Medicine, University of Lagos, Idi-Araba, Lagos, Nigeria

**Keywords:** Malaria rapid diagnostic test, Inverted cup blood transfer device, Ease of use, Safety and acceptability

## Abstract

**Background:**

Malaria rapid diagnostic tests (RDTs) are becoming widely adopted for case management at community level. However, reports and anecdotal observations indicate that the blood transfer step poses a significant challenge to many users. This study sought to evaluate the inverted cup device in the hands of health workers in everyday clinical practice, in comparison with the plastic pipette, and to determine the volume accuracy of the device made of a lower-cost plastic.

**Methods:**

The volume accuracy of inverted cup devices made of two plastics, PMMA and SBC, was compared by transferring blood 150 times onto filter paper and comparing the blood spot areas with those produced by 20 reference transfers with a calibrated micropipette. The ease of use, safety and acceptability of the inverted cup device and the pipette were evaluated by 50 health workers in Nigeria. Observations were recorded on pre-designed questionnaires, by the health workers themselves and by trained observers. Focus group discussions were also conducted.

**Results:**

The volume accuracy assessment showed that the device made from the low-cost material (SBC) delivered a more accurate volume (mean 5.4 μL, SD 0.48 μL, range 4.5–7.0 μL) than the PMMA device (mean 5.9 μL, SD 0.48 μL, range 4.9–7.2 μL). The observational evaluation demonstrated that the inverted cup device performed better than the pipette in all aspects, e.g. higher proportions of health workers achieved successful blood collection (96%, vs. 66%), transfer of the required blood volume (90%, vs. 58%), and blood deposit without any loss (95%, vs. 50%). Majority of health workers also considered it’ very easy’ to use (81%),’very appropriate’ for everyday use (78%), and 50% of them reported that it was their preferred BTD.

**Conclusions:**

The good volume accuracy and high acceptability of the inverted cup device shown in this study, along with observed ease of use and safety in hands of health workers, further strengthens prior findings which demonstrated its higher accuracy as compared with other BTDs in a laboratory setting. Altogether, these studies suggest that the inverted cup device should replace other types of devices for use in day-to-day malaria diagnosis with RDTs.

## Background

Malaria rapid diagnostic tests (RDTs) are becoming widely used and adopted into country program protocols for use in case management in public health facilities and at community level. According to the World Malaria Report, the sales of malaria RDTs increased dramatically, from an estimated 50 million in 2008 to 314 million in 2014 [1]. This is due to their use in areas where good quality microscopy is not available, including peripheral health centres and after-hours at larger facilities. They are used by large numbers of personnel with minimal or no training in laboratory techniques. Studies have demonstrated that health workers with minimal formal training can satisfactorily perform and interpret RDTs [2–4].

Most commercially available RDT kits are packaged with individual-use disposable blood transfer devices (BTDs) that are used to collect, transfer and deposit a specific amount of blood from a finger prick site to a well on the RDT cassette. Reports and anecdotal observations have repeatedly indicated that blood transfer is an aspect of RDT use that poses a significant challenge to many users [5–8]. Typical concerns are that they may raise the risk of blood exposure, they may not reliably transfer an appropriate amount of blood (leading to risk of false-negative results or difficult result interpretation because of a strong red background) and they may be difficult for many health workers to manipulate [5].

**Fig. 1:**
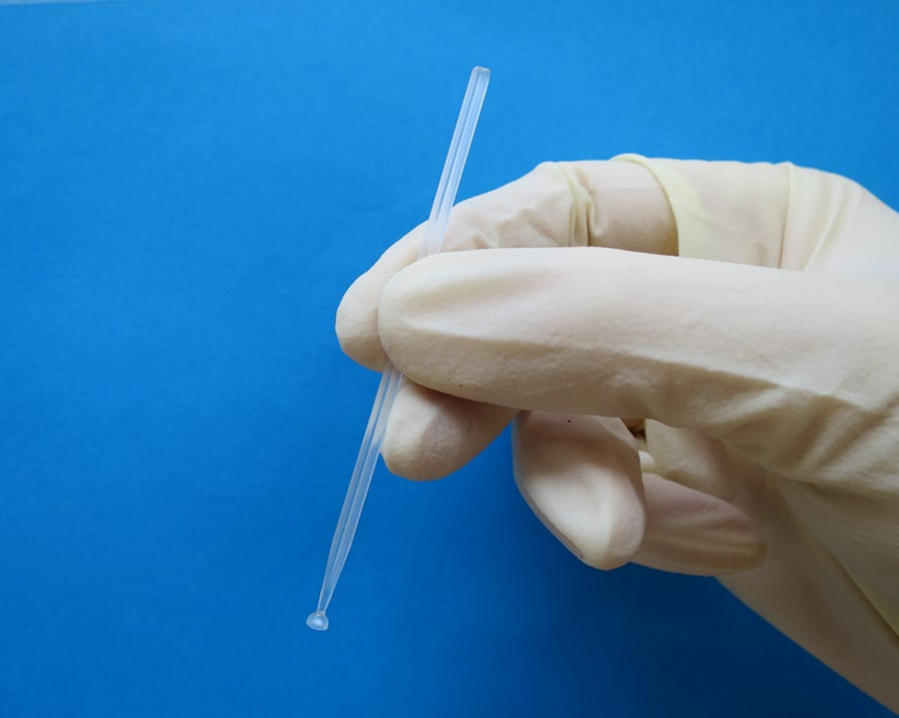
Inverted cup blood transfer device

In 2011, a study identified the inverted cup device (Fig. 1) as the most appropriate blood transfer device for use with malaria RDTs, among a list of 5 different devices, not only in terms of the blood volume accuracy, but also in terms of ease of use and acceptability [9]. The inverted cup BTD is an individual disposable plastic device with an inverted cup shape at the base of a narrow stem, specifically designed for reliable uptake and release of whole blood as it immediately fills up with the adequate volume of blood when held in contact with a blood drop, and similarly easily drains the entire volume when put in contact with the filter pad of a malaria RDT. Since the design became publicly available, this device has been adopted successfully by various manufacturers, with more than 130 million units having been distributed in 2015 (FIND, unpublished data). The previous study was undertaken with health workers performing transfers of anti-coagulated blood from a mimicked fingerprick only, therefore, the use of this BTD in a real point-of-care setting with febrile patients had not been evaluated to date. The primary goal of the present study was to assess the ease of use and the safety of the inverted cup BTD in the hands of health workers in Nigeria in the context of their daily patient care activity, and to demonstrate its acceptability for use, in comparison with the standard device (i.e. pipette) provided with RDT kits being used at that time in Nigerian public health facilities.

In addition to this, a laboratory assessment of inverted cup devices made from two different plastic materials was conducted, in order potentially to identify a low-cost option for the production of the device while still maintaining performance. The initial material used in the manufacture of the inverted cup device, polymethylmethacrylate (PMMA), was effective, but expensive, so a low-cost material, styrene butadiene copolymers (SBC), was identified as a potential alternative, costing nearly half the price of the PMMA. Inverted cup devices made of the two different plastics, but identical in design, were compared in terms of the accuracy of blood volumes transferred.

## Methods

### Comparison of volume accuracy of inverted cup devices made of PMMA and SBC

To measure the accuracy and consistency of the volume of blood collected and transferred using the PMMA and SBC inverted cups, an assessment was carried out at the Parasitology Reference Laboratory, Department of Clinical Parasitology, Hospital for Tropical Diseases, London, UK, where microscopy is available for malaria diagnosis and RDTs are used in addition by laboratory staff on-call at night.

A single sample of venous blood from a volunteer blood donor, pre-screened for blood-borne infectious agents, was provided in an ethylenediaminetetraacetic acid (EDTA) tube. Using a micropipette, 5 μL aliquots of blood were spotted 20 times onto Whatman grade 3MM filter paper and air-dried. A small volume of blood (e.g. 50 μL) was first transferred to a gloved fingertip. Each of the two BTDs was then used to transfer blood from this simulated fingerprick to filter paper. This was repeated using a new device each time until 150 blood spots were obtained for both study devices.

The blood transfers were all performed by a single technician researcher using the same tube of venous blood, given that variations in blood haematocrit, viscosity, ambient temperature, humidity and other factors could influence the absorptive capacity of filter paper and consequently the size of blood spots.

The area of each dried blood spot was then calculated using dedicated software (LineScale V180, LineType Software, Inc.) by another investigator, and the mean values as well as standard deviations determined for each of the three transfer types (micropipette, PMMA BTD and SBC BTD). The mean area of the 20 blood spots produced using the micropipette was used to calculate a standard area-to-volume coefficient that was subsequently used to estimate the volume of blood transferred by the two inverted cup BTDs.

All data were entered into MS Excel 2010, verified and then transferred into SAS version 9.3 (SAS Institute Inc., USA) for comparison of results obtained using the two devices with each other, as well as with the values obtained from the reference blood spots produced using the micropipette.

### Ease of use, safety and acceptability of the inverted cup device and the pipette in the hands of health workers in Nigeria

#### Study population

This study took place from March to June 2012 among 50 health workers from 20 Primary Health Centres in Lagos, Nigeria. Parasite-based diagnosis of all suspected malaria cases has been the Nigerian policy for malaria diagnosis and treatment since 2010. Health centres were selected based on high turnover of malaria patients, routine use of malaria RDTs in malaria case management, proximity to the College of Medicine, University of Lagos, Idi-Araba, Lagos, willingness to collaborate, and a minimum of 1 and up to 4 health workers willing to participate in the study. The health workers were selected on the basis of staff membership of the participating health facility in a patient-care role, minimal experience with malaria RDTs and written consent to participate in the study. The study was ethically approved by the Ministry of Health of Lagos State, Nigeria.

#### Training of health workers

The SD Bioline Malaria Ag Pf device RDT kits (catalog number 05FK50, Standard Diagnostics Inc., South Korea) were used in this study, as being the RDT kits routinely distributed in the Nigerian public health facilities at the time of the study. Health workers received a brief standardized training and a job aid on the correct use of the inverted cup device. They then had the opportunity to practice, a maximum of five times, blood transfer to two malaria RDTs using the inverted cup device for one, and the pipette, as being the standard device included in the SD Bioline Malaria Ag Pf kits, for the other. A single sample of venous blood from a volunteer blood donor, pre-screened for blood-borne infectious agents was provided in an EDTA tube. For each blood transfer exercise, 10 μL of blood was deposited on a plain latex surface, from which the health worker then transferred 5 μL to a malaria RDT cassette using the inverted cup device, and the same to another cassette using the pipette. Any questions and difficulties were discussed during and at the end of the training period.

#### Health workers use of BTDs

Presenting patients of all ages having symptoms of malaria were invited to participate in the study, and included if they provided written informed consent. Each health worker performed malaria RDTs on 10 patients. Transfer of approximately 5 µL of finger prick blood was performed with each of the two devices, from the same finger prick of each patient. There was no particular instruction for the order of use of BTD, and the health workers arbitrarily chose which device to use first and second. The RDT result obtained with the standard transfer device (pipette) was communicated to the patient as per routine clinical practice, regardless of the result obtained with the test based on transfer with the inverted cup device. Patients with a positive result were treated according to the national Nigerian treatment guidelines with artemether-lumefantrine combination treatment.

#### Assessment of ease of use, safety and acceptability

Members of the study team observed the health workers during each step of the blood transfers for each of the 10 patients, and completed a standardized observational checklist comprising questions about the blood collection, transfer, blood deposit, and blood spillage. Once the health worker had completed the work with 10 patients, he/she was asked to complete a standardized questionnaire to evaluate his/her perception of ease of use, risk of blood exposure, acceptability, and preference of BTDs.

### Data analysis

Data from the laboratory study of the PMMA and the SBC inverted cup devices were entered into Microsoft Office Excel 2010. The overall mean, range and standard deviation of blood spot areas and converted volumes were calculated for each BTD, taking each attempt as the unit of analysis. The Shapiro–Wilk test was performed on the three groups to confirm normality using Stata I/C 11.1 (StataCorp LP, USA). Random Effects GLS regression was then performed as the test of independence using Stata I/C 11.1. Levene’s test for homogeneity indicated heteroscedasticity (unequal variances) across groups at the α = 0.05 level of significance. Mean values and standard deviations (SD) of blood spot areas for the micropipette, SBC and PMMA were therefore compared with each other (e.g. micropipette-SBC; micropipette-PMMA; SBC-PMMA) using least squares means (LSMEANS In SAS) with Dunnett’s modified tukey–kramer adjustment or T3 method in SAS 9.3. This accounted for unequal variance and unequal sample size across groups as is the case in the reference group compared to the two BTDs [10].

Data from the field study with health workers in Nigeria were collected using pre-designed questionnaires, one for recording the observations from the study observers, and one for recording perceptions of the health workers. A majority of questions allowed for a categorization of answers, either as 1-Yes or 2-No, or with rates from 1 to 5 with meanings of each rate being explained e.g. 1-very easy to 5-very difficult. Some questions also allowed for free expression of opinions or suggestions. The categorized answers were coded and then recorded with double data entry using Epi Info, 2002 (Centers for Disease Control and Prevention, Atlanta, Georgia, USA). Analysis was done using Microsoft Office Excel 2010.

## Results

### Comparison of volume accuracy of inverted cup devices made of PMMA and SBC

The mean area for the reference group of blood spots produced using a micropipette was 21.4 mm^2^ (SD 1.1 mm^2^). Spots produced with the inverted cup BTDs had mean areas of 23.0 and 25.4 mm^2^, with the same standard deviation of 2.1 mm^2^, when using the SBC and PMMA plastic devices, respectively.

The area–volume conversion coefficient was calculated by dividing the mean area of the reference blood spots (21.4 mm^2^) by the reference volume of 5 μL, obtaining a coefficient of 4.3. Using this coefficient, the mean volumes and standard deviations delivered by the two inverted cup BTDs made of SBC and PMMA were 5.4 μL (SD 0.48 μL, minimum 4.5 μL, maximum 7.0 μL) and 5.9 μL (SD 0.48 μL, minimum 4.9 μL, maximum 7.2 μL), respectively.

The differences between the mean blood spot areas produced by each of the three transfer types were all statistically significant, when comparing them two by two (P < 0.0001 in all cases), as per least squares means (LSMEANS In SAS) with Dunnett’s modified Tukey–Kramer adjustment or T3 method.

**Fig. 2:**
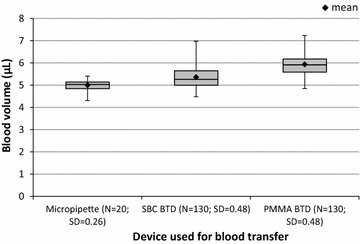
Volume of blood deposited on filter paper using a micropipette versus inverted cup BTDs. Box plot diagrams showing the blood volume, in µL, deposited on filter with a calibrated micropipette (set volume of 5 µL), and with inverted cup blood transfer devices (BTDs) made of two different plastics (*SBC BTD* inverted cup BTD made of styrene butadiene copolymers, *PMMA BTD* inverted cup BTD made of polymethylmethacrylate). *N* number of blood deposits, *SD* standard deviation

The mean volume transferred with the SBC inverted cup device (5.4 μL) is therefore significantly closer to the reference volume of 5 μL than the volume transferred with the PMMA inverted cup device, hence the use of this low-cost plastic for manufacturing inverted cup devices is acceptable (Fig. 2).

### Ease of use, safety and acceptability of the inverted cup device and the pipette in the hands of health workers in Nigeria

#### Prior health worker experiences with BTDs

72% (39/54) of the health workers had prior experience with RDTs and BTDs. Of those who had used BTDs before, the devices used were the pipette (52%), the glass capillary (24%), the inverted cup (21%) and the loop (3%). Of the 30 health workers who responded if their prior experiences were positive or negative, 70% reported a negative experience, with the main reasons being that BTDs were ‘difficult’ or ‘very difficult’ to use. Specific to the pipette, the health workers reported that it was difficult to handle, made work slower and decreased confidence in front of the patient. There were no complaints specific to the inverted cup. In general, the health workers suggested that more training on proper use of the RDTs with BTDs would be required.

#### Objective evaluation by observers

**Fig. 3:**
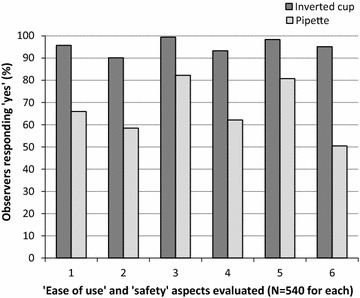
Observation of health workers doing blood transfers with the inverted cup BTD and the pipette. 1 = successful blood collection in one attempt; 2 = the device was appropriately filled with blood; 3 = blood was not unintentionally released from BTD before reaching RDT; 4 = successful blood deposit in one attempt; 5 = blood did not touch the health workers gloves, skin or clothing at any time; 6 = no blood remaining in the BTD after deposit in the RDT well. *N* number of observations

The observation of health workers during the different steps of blood transfer showed much better results for the inverted cup, as compared to the pipette, in all the 6 aspects of ease of use or safety that were used to evaluate the devices (Fig. 3). The success rates for the inverted cup ranged from 90% observations of a successful filling of the cup with blood, up to 99% observations of transfers without any unintentional release of blood. For the pipette, rates were lowest for transfers without any blood remaining in the BTD (50%), meaning that less blood than desired was deposited on the RDTs in half of the cases. Overall, the devices performed better on the two aspects related to blood safety (aspects no. 3 and 5), while aspects related to good blood uptake (aspects no. 1 and 2) and proper blood deposit (aspects no. 4 and 6) appeared to be more critical. The difference between the two devices was most striking when looking at the blood remaining in the BTD after deposit on the RDT (95% success rate for the inverted cup versus only 50% for the pipette).

#### Health worker perspectives

**Fig. 4:**
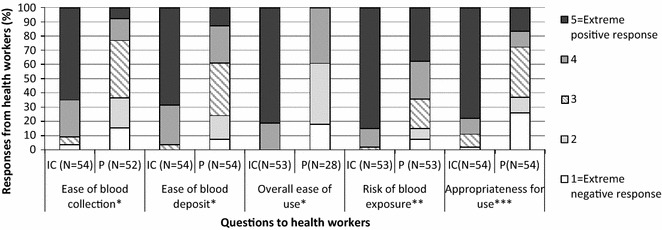
Health worker opinions on the inverted cup BTD and the pipette.*1 = very difficult; 2 = not easy; 3 = little bit easy; 4 = easy; 5 = very easy. **1 = great risk; 2 = quite some risk; 3 = little risk; 4 = very little risk; 5 = no risk. ***1 = not appropriate; 2 = little appropriate; 3 = manageable; 4 = appropriate; 5 = very appropriate. *IC* inverted cup BTD, *P* pipette, *N* number of observations

**Fig. 5:**
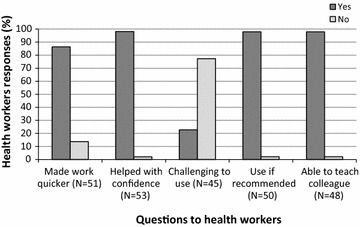
Health worker perceptions on the inverted cup BTD. Health workers responses when being asked different questions about the inverted cup BTD. *N* number of observations

**Fig. 6:**
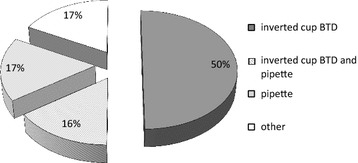
Preferred blood transfer device. Preference of health workers for various options of blood transfer devices, shown as a percentage of a total of 52 health workers having been asked

The appreciation of health workers mirrored the results from the observational study, with much higher rates of positive opinions about the inverted cup device, as compared to the pipette, for all five aspects on which the health workers were questioned (Fig. 4). A majority of health workers found the inverted cup device ‘very easy’ to use in terms of ease of blood collection by comparison with the pipette (65% inverted cup; 8% pipette), ease of blood deposit (69%; 13%) and overall ease of use (81%; 0%), and a majority considered there was ‘no risk’ of blood exposure (85%; 38%), and that it was ‘very appropriate’ for use in every day clinical work (78%; 17%). The differences between the inverted cup device and the pipette are even higher when asking health workers about a number of perceptions (Fig. 5), e.g. a large majority of health workers reported that the device made their work quicker (inverted cup 86%; pipette 14%), helped with their confidence in front of the patient (98%; 2%), that they would use it if recommended (98%; 2%) and that they were able to teach a colleague how to use it (98%; 2%). When asked about their preferred BTD, 50% reported that it was the inverted cup (Fig. 6).

## Discussion

The purpose of this study was to gather evidence on the appropriateness and benefit of using the inverted cup blood transfer device for performing malaria RDTs in a point-of-care setting. In addition, a small laboratory study compared two different plastics, in order to evaluate the performance of a second inverted cup device made from a lower-cost plastic, hereby offering an alternative option for reduced manufacturing costs.

The use of different plastics modifies the liquid adherence characteristics of the little cup, hence potentially modifying the retention properties of the device, and in turn, the volume of blood that is being collected and then delivered upon the contact with the RDT filter pad. Since the blood volume is critical, substandard volumes will potentially produce substandard RDT results, i.e. high red background because of too much blood, or faint test lines or false negative results because of too little blood. The results of this study are highly valuable, since they show that the SBC (lower cost plastic) inverted cup device in fact dispenses a volume of blood that is even closer to the required reference volume of 5 μL, with the same standard deviation as the PMMA device.

It was felt important that the inverted cup device, previously identified as the preferred device among a list of 5 different BTDs, could be included in RDT kits by RDT manufacturers without increasing the costs of the kit overall, and -ideally-even decrease the kits’ cost if the inverted cup device proves cheaper than other BTDs. For purposes of cost comparison, all factors remaining constant, the average unit price of an inverted cup device made of PMMA is 0.0115 euros, compared with 0.0064 euros for a device made of SBC (Injection 74, Alex, France, personal communication). The field study presented in this report was based on BTDs made of the low-cost material SBC.

This is the first study evaluating the inverted cup BTD in the hands of health workers in everyday clinical practice. A majority of the Nigerian health workers enrolled for this study had used BTDs before and not only for malaria diagnosis. Though a number had used various BTDs on the market, a majority had used the pipette.

Collecting, or picking up, blood from a patient finger prick into the BTD is the first step of the transfer process. Using a pipette transfer device, this step can prove difficult, because it requires maintaining a constant and adequate suction pressure on the squeezable part of the pipette; a health worker also noted formation of bubbles as a challenge with this device. With the inverted cup device however, simple contact of the cup with the blood drop is sufficient for the blood to be automatically drawn and fill the cup. These inherent differences between the two devices probably explain the study findings where a majority of the health workers was able properly to collect the blood in only one attempt with the inverted cup device, while more than one attempt was often needed to do so with the pipette.

After blood collection, it is prudent to verify if the devices collected the required amounts of blood, to prevent the risk of test results with a high red background (if too much blood) or faint test lines, or even false negative results (if not enough blood). For the pipette or capillary devices, this involves filling the device to the given titration markings. Unfortunately, these are not always easily seen-especially if there is no proper lighting, or health workers do not pay the required attention because of time pressure, or in some cases they are not even aware that the blood volume is so critical. Other devices, such as the loop and the inverted cup are pre-designed to pick up exactly the required amount of blood. Considering that blood transfer devices are used for point of care diagnosis in lower level facilities, devices that automatically pick up the specific amount of blood clearly offer an added advantage. The results of this study confirm the above considerations about ease of use, since a majority of the health workers were able to fill completely the inverted cup, as opposed to only a minority being able to fill the pipette to the calibrated markings.

The observational study also looked at the ease of depositing blood and at blood remaining in the device afterwards. Again, the study shows much better results for the inverted cup device than for the pipette (95% vs. 50% of successful deposits without any blood remaining in the device). While using the pipette, the health worker is required to apply adequate pressure to deposit the blood successfully. Accidental release of that pressure introduces bubbles, and has the reverse effect of the blood being sucked deeper into the device rather than being released onto the RDT. For the inverted cup, simple contact of the blood drop with the filter pad leads to an automatic release and absorption of the blood by capillary action. As for the blood collection, it is again the inherent characteristics of the two devices that most probably explain the much higher success rates of the inverted cup device.

The differences between the two devices were less marked regarding blood safety, yet the inverted cup device again performed better than the pipette. The blood safety aspect involves unintentional release or spillage of blood, with resultant contamination of health workers’ gloves, skin, clothing or surfaces at any time during the transfer. Blood safety issues with the pipette are often due to unintentional pressure on the squeezable part of the pipette during the transfer, resulting in blood spillage, while blood exposure with the inverted cup device might happen when unintentionally touching any surface, which is a less common risk.

Not surprisingly, the results of the health workers’ perceptions study mirror those of the observational study, confirming that health workers feel more comfortable with every step of the blood transfer when using the device that performs better. Results show that the inverted cup is perceived as being more appropriate for everyday use than the pipette, easier to handle, making work quicker and helping more with confidence in front of the patient. Health workers also reported that they would use the device if recommended for use, and that they would be able to teach their colleagues how to use it.

One limitation of this study is that the inverted cup device is compared here only to the pipette device, as being the device included in the RDT kits provided to public health facilities in Nigeria at the time of the study, hence differences with other devices such as the loop or the capillary are not assessed. Based on the previously published study that compares five different devices, the loop device would be a potentially valuable alternative, with that study nevertheless pointing to the inverted cup as the preferred one.

As mentioned above, an estimated 130 million inverted cup devices were distributed in RDT kits in 2015, showing that the benefit of using the inverted cup device has already been recognized by RDT manufacturers. However, less practical and less well-performing BTDs (such as the pipette), are still being used with various RDT products. More communication with regard to the inverted cup device would obviously contribute to an increased uptake, and in turn, given the results of the perception study reported here, contribute to improved confidence of RDT users. Published reports show that despite wide implementation of RDT diagnosis, there is still a lack of confidence in the quality of RDTs and confidence in test results, leading to unnecessary treatment of test-negative patients with anti-malarials [11–13]. Deployment of an easier to use blood transfer device like the inverted cup might be one of the changes that could contribute to improved confidence in RDTs overall, by responding to the challenge that health workers face with BTD manipulation.

Proper training on the use of blood transfer devices is another important aspect to resolve difficulties in the use of RDTs. The advantage of the inverted cup device is that it does not require fine handling such as the squeezable pipette for example. It is however important to emphasize that the device should always be held in a vertical position, both for blood collection and even more so for blood deposition. Generally, it is recommended that training on the use of RDTs should always include dedicated time for explaining the use of the various BTDs that are available in commercial RDT kits, preparing health workers for potential changes to RDT products, including BTDs. Training manuals including specific sections on the use of blood transfer devices have been developed and field-tested [14], and a troubleshooting guide with extra-pages focusing on the use, potential difficulties and recommended solutions for 5 different devices is publicly available [15].

## Conclusions

This study confirms findings of previously published studies showing that the inverted cup blood transfer device performs better than other devices, focusing here on the context of health workers in routine clinical care testing patients presenting with symptoms of malaria. The study results suggest that replacement of the plastic pipette and other blood transfer devices by the inverted cup device would facilitate the use of RDTs in point-of-care settings, contributing to improved confidence in RDTs for the diagnosis of malaria. The inverted cup device can be manufactured with a low-cost material, SBC plastic, without adversely affecting performance and accuracy of blood volume transfer.

## Abbreviations

BTD: blood transfer device
RDT: rapid diagnostic test
SBC: styrene-butadiene copolymers
PMMA: polymethylmethacrylate
EDTA: ethylenediaminetetraacetic acid
FIND: Foundation for Innovative New Diagnostics
FMoH: Federal Ministry of Health
